# Supplementary material for: Predicting visual function by interpreting a neuronal wiring diagram
Source: Nature. 2024 Oct 2;634(8032):113–23. doi: 10.1038/s41586-024-07953-5 (PMC11446822; doi:10.1038/s41586-024-07953-5)
Supplement: Supplementary file 6 — CRF and ERF predictions for individual TmY4 and TmY9 cells. Analogous to Supplementary Data 3, but for TmY target types. Shown are the top four monosynaptic pathways, the strongest pathway passing through each of the top ten intermediary types (ranking from Extended Data Fig. 7), and the trisynaptic pathway Tm1–TmY–Dm3–TmY (see the section entitled Prediction of spatial normalization). [file 41586_2024_7953_MOESM6_ESM.zip › DataS4/TmY9q/720575940627891880.pdf]

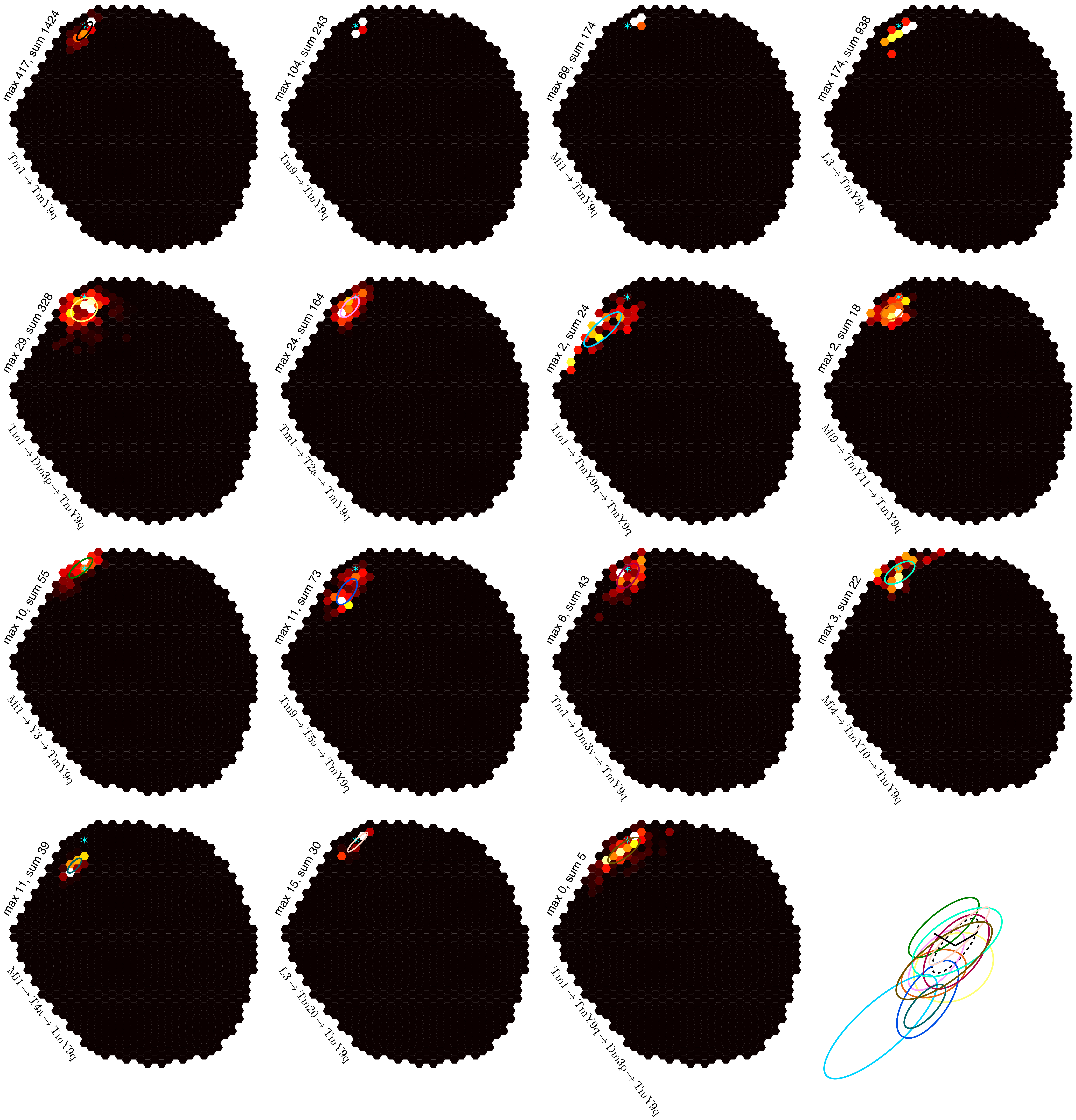

max 417, sum 1424
Tm1 → TmY9q
max 104, sum 243
Tm9 → TmY9q
max 69, sum 174
Mi1 → TmY9q
max 174, sum 938
L3 → TmY9q
max 29, sum 328
Tm1 → Dm3p → TmY9q
max 24, sum 164
Tm1 → T2a → TmY9q
max 2, sum 24
Tm1 → TmY9q → TmY9q
max 2, sum 18
Mi9 → TmY11 → TmY9q
max 10, sum 55
Mi1 → Y3 → TmY9q
max 11, sum 73
Tm9 → T5a → TmY9q
max 6, sum 43
Tm1 → Dm3v → TmY9q
max 3, sum 22
Mi4 → TmY10 → TmY9q
max 11, sum 39
Mi1 → T4a → TmY9q
max 15, sum 30
L3 → Tm20 → TmY9q
max 0, sum 5
Tm1 → TmY9q → Dm3p → TmY9q
